# Influenza-Specific Antibody-Dependent Phagocytosis

**DOI:** 10.1371/journal.pone.0154461

**Published:** 2016-04-28

**Authors:** Fernanda Ana-Sosa-Batiz, Hillary Vanderven, Sinthujan Jegaskanda, Angus Johnston, Steven Rockman, Karen Laurie, Ian Barr, Patrick Reading, Marit Lichtfuss, Stephen J. Kent

**Affiliations:** 1 Department of Microbiology and Immunology at the Peter Doherty Institute for Infection and Immunity, The University of Melbourne, Melbourne, Australia; 2 Drug Delivery, Disposition and Dynamics laboratory, Monash Institute of Pharmaceutical Sciences, Monash University, Melbourne, Australia; 3 ARC Centre of Excellence in Convergent Bio-Nano Science and Technology, Monash University, Parkville, Australia; 4 bioCSL Ltd, Parkville, Victoria, Australia; 5 WHO Collaborating Centre for Reference and Research on Influenza at the Peter Doherty Institute for Infection and Immunity, Melbourne, Australia; 6 Melbourne Sexual Health Centre, Central Clinical School, Monash University, Melbourne, Australia; 7 ARC Centre of Excellence in Convergent Bio-Nano Science and Technology, University of Melbourne, Parkville, Australia; Icahn School of Medicine at Mount Sinai, UNITED STATES

## Abstract

**Background:**

Immunity to human influenza A virus (IAV) infection is only partially understood. Broadly non-neutralizing antibodies may assist in reducing disease but have not been well characterized.

**Methods:**

We measured internalization of opsonized, influenza protein-coated fluorescent beads and live IAV into a monocytic cell line to study antibody-dependent phagocytosis (ADP) against multiple influenza hemagglutinin (HA) subtypes. We analyzed influenza HA-specific ADP in healthy human donors, in preparations of intravenous immunoglobulin (IVIG), and following IAV infection of humans and macaques.

**Results:**

We found that both sera from healthy adults and IVIG preparations had broad ADP to multiple seasonal HA proteins and weak cross-reactive ADP to non-circulating HA proteins. The ADP in experimentally influenza-infected macaque plasma and naturally influenza-infected human sera mediated phagocytosis of both homologous and heterologous IAVs. Further, the IAV phagocytosed in an antibody-mediated manner had reduced infectivity *in vitro*.

**Conclusion:**

We conclude that IAV infections in humans and macaques leads to the development of influenza-specific ADP that can clear IAV infection *in vitro*. Repeated exposure of humans to multiple IAV infections likely leads to the development of ADP that is cross-reactive to strains not previously encountered. Further analyses of the protective capacity of broadly reactive influenza-specific ADP is warranted.

## Introduction

There is a need to better understand immunity to influenza A viruses (IAVs) to rationally develop improved vaccines. [1–5]. Neutralizing antibodies (NAbs) can prevent influenza virus infection (IVI) but are primarily directed towards the highly variable regions of the surface hemagglutinin (HA) protein of influenza and are frequently strain specific. There is great interest in NAbs to the conserved stalk regions of HA. Strategies to induce such antibodies by vaccination are under development [6, 7].

Infection and/or vaccination can lead to the induction of a range of functional antibodies. Non-neutralizing antibodies (non-NAbs) have the potential to bind conserved regions of HA. Antibody-dependent cellular cytotoxicity (ADCC) triggering antibodies bind to viral proteins on the surface of infected cells, and engage the CD16 Fc receptor [8, 9] to activate innate immune cells [10–12]. ADCC triggering antibodies induced by serial influenza infections of humans are frequently cross-reactive [10, 13, 14] and are important in protecting mice from IAV [15, 16].

Non-NAbs can also mediate antibody-dependent phagocytosis (ADP) through CD32, CD64 and CD89 receptors [8]. ADP plays a role in the prevention of HIV infection, where a composite of non-NAb functions appears to contribute to the partial efficacy of a HIV vaccine candidate [17]. There is one report that influenza infection of mice can generate antibodies that mediate ADP but this has not been explored further [18].

In this report we both adapted a HIV-specific ADP assay [19] to measure influenza-specific ADP and also developed an *in vitro* functional assay that measures antibody-mediated clearance of live IAVs. We assessed sera from humans and macaques infected with influenza to determine the potential of HA-specific ADP antibodies.

## Materials and Methods

### Influenza ADP-SHIP assay

**Fig 1:**
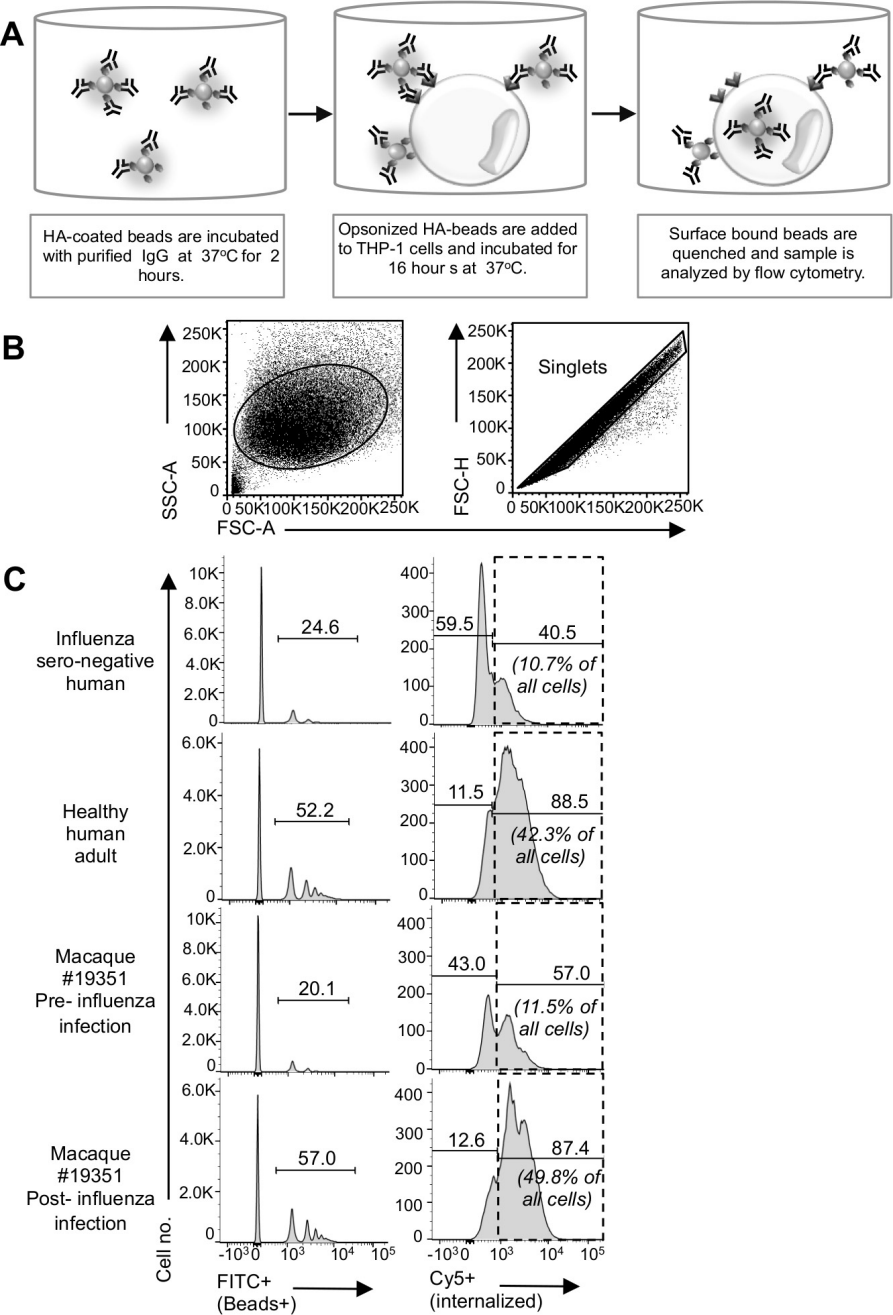
Representative influenza HA-specific ADP assay. The ADP assay is performed as describe in **(A).** In brief, FITC fluorescent beads coated with hemagglutinin (HA) and fluorescent internalization probe (FIP_Cy5_) are opsonized with 10μg/ml purified IgG from blood plasma or sera. HA-coated beads are incubated 16 hr with monocyte-like cell line, THP-1. Cy5 fluorescence from surface bound beads is quenched. Cells are fixed and analysed by flow cytometry. **(B)** Gating strategy of single cells. **(C)** Gating strategy for internalized beads+ (FITC+, Cy5+) and surface associate beads+ (FITC+. Cy5-) cells. Uptake of H3N2 A/Wyoming/03/2003 HA-coated beads in presence of influenza sero-negative human serum IgG or healthy human adult plasma IgG (top 2 panels). Uptake of H1N1 A/Puerto Rico/08/1939 HA-coated beads opsonized with IgG from macaque plasma either pre-influenza infection or post-influenza infection (bottom 2 panels). Data are representative of 14 human donors (Fig 2) and 6 macaques (Fig 5).

ADP was performed by measuring the internalization of opsonized HA-coated beads by a phagocytic cells line (Fig 1A). The methods were similar to those previously described for HIV [19, 20] with minor modifications. Briefly, FITC-labeled NeutrAvidin^®^ FluoSpheres^®^ (beads 1μm, Invitrogen, Carlsband, CA) were labeled both with internalization probe tagged with Cy5 (FIP_Cy5_) [21] and 0.75μg biotinylated HA or HIV-1 gp140 then opsonized with 10μg/ml purified IgG (Protein G HP Multitrap, GE Healthcare, UK). 1x10^5^ THP-1 (ATCC TIB-202) cells were incubated with the beads for 16 hr. A 16hr incubation provided a reasonable balance between ADP and non-specific bead uptake (not shown). Cell surface FIP_Cy5_ was quenched with a complimentary probe so that internalized beads (FITC+Cy5+—i.e. truly phagocytosed) can be measured. Cells were fixed and 5x10^4^ cells were analyzed by flow cytometry. Background levels of ADP activity were assessed against HIV-1 gp140 since all donors were HIV-negative and calculated as the mean plus 2 SD. Background ADP levels were reproducible across multiple experiments (11–14%) as illustrated in the dotted lines Figs 1B and 3A–3C.

**Fig 2:**
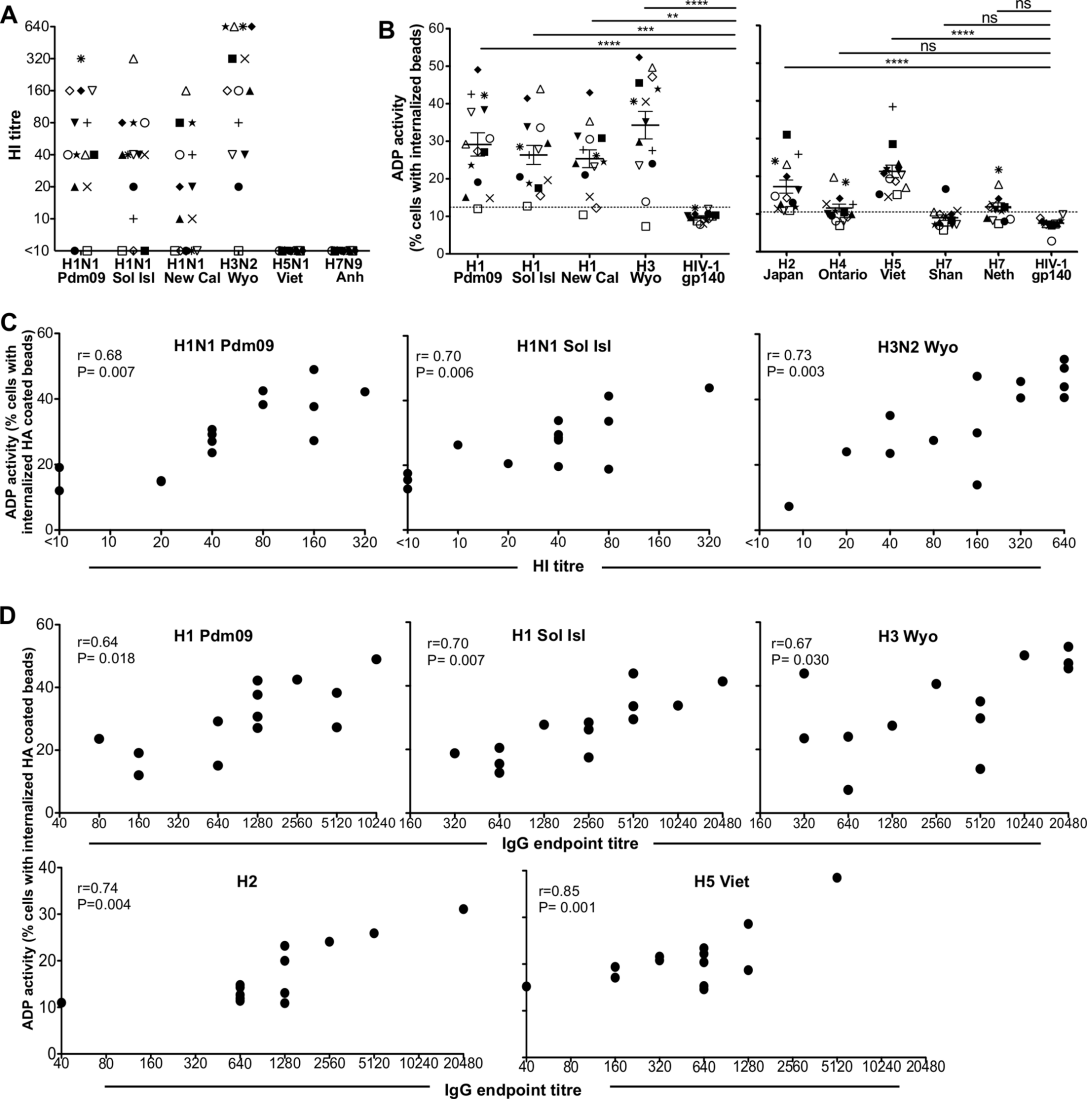
Comparison of HI titres and ADP activity in healthy adults. **(A)** H1N1 A/California/04/2009 (H1N1 Pdm09), A/Solomon Islands/03/2006 (H1N1 Sol Isl), A/New Caledonia/20/1999 (H1N1 New Cal), H3N2 A/Wyoming/03/2003 (H3N2 Wyo), H5N1 A/Vietnam/1194/2004 (H5N1 Viet) γ-irradiated and H7N9 A/Anhui/01/2013 (H7N9 Anh) γ-irradiated hemagglutination inhibition (HI) titres were determined for 14 healthy donors. **(B)** ADP activity in serum IgG against seasonal H1N1 and H3N2 HAs **(left panel)** and rare H2N2 A/Japan/503/1957 (H2 Japan), H4H6 A/Swine/Ontario/01911-1/1999 (H4 Ontario), H5N1 A/Vietnam/1194/2004 (H5 Viet), H7N9 A/Shanghai/1/2013 (H7 Shan) and H7N7 A/Netherlands/219/2003 (H7 Neth) HAs **(right panel)** was evaluated. HIV-1 gp140 was included as negative control in each experiment. Line and error bars represent mean ± SEM. **** P < 0.0001, *** P < 0.001, ** P < 0.01, and ns P > 0.05 determined using Bonferroni’s multiple comparison test. Dotted line indicated mean + twice SD of ADP for HIV-1 gp140. **(C)** Correlation of H1 Pdm09, H1 Sol Isl and H3 Wyo HA-specific ADP activity and HI titre to the same virus determined using Pearson’s correlation. Data are representative of n = 3 independent experiments. **(D)** Correlation of H1 Pdm09, H1 Sol Isl, H3 Wyo, H2 and H5 Viet HA-specific ADP activity and IgG endpoint tires to the same or similar virus using Pearson’s correlation. Serial dilutions of plasma on IgG ELISA were performed in duplicate.

**Fig 3:**
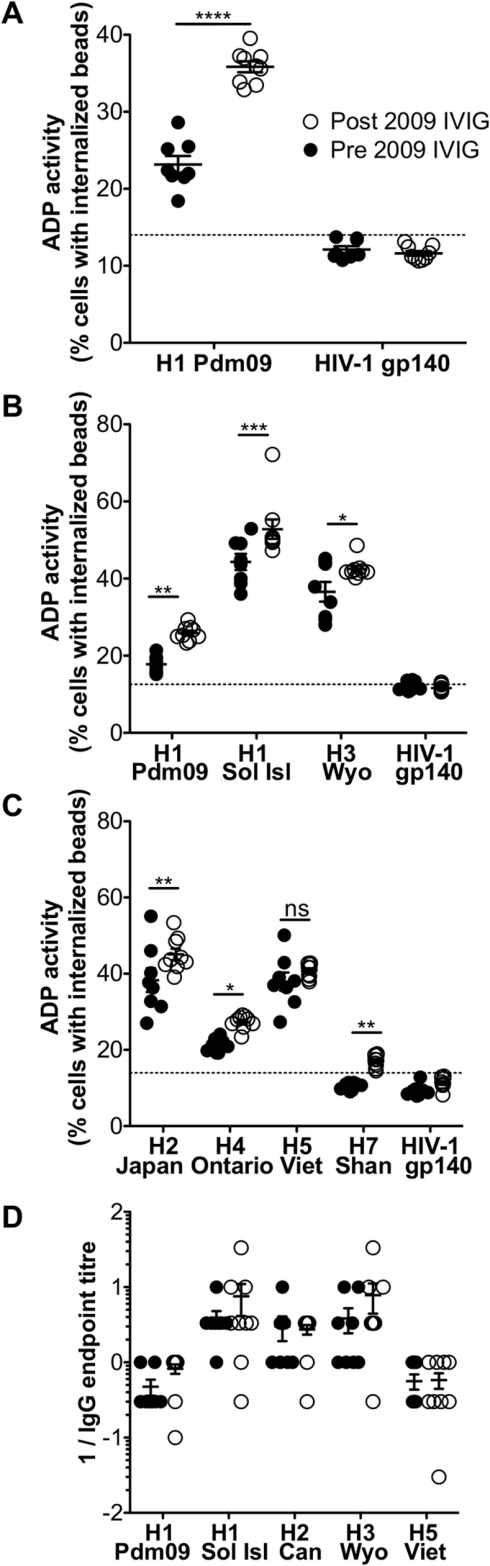
Assessment of ADP activity in IVIG preparations. **(A)** ADP activity against H1 Pdm09 HA was determined for 8 pre-2009 and 9 post-2009 IVIG preparations. Differences in ADP activity comparing pre and post 2009 IVIG samples were evaluated against seasonal H1 and H3 HAs **(B)** and non-circulating H2N2 A/Japan/503/1957 (H2 Japan), H4H6 A/Swine/Ontario/01911-1/1999 (H4 Ontario), H5N1 A/Vietnam/1194/2004 (H5 Viet) and H7N9 A/Anhui/1/2013 (H7 Shan) HAs **(C)**. HIV-1 gp140 was included in each experiment as negative control. Dotted line indicated mean + twice SD of ADP for HIV-1 gp140. Line and error bars represent mean ± SEM. **** P < 0.0001, *** P < 0.001, ** P < 0.01, * P < 0.05 and ns P > 0.05 determined using Bonferroni’s multiple comparison test. Data are representative of n = 3 independent experiments. **(D)** IgG endpoint titres were calculated for each IVIG sample to H1 Pdm09, H1 Sol Isl, H3 Wyo, H2 and H5 Viet. Half-log serial dilutions ranging from 100 to 0.030 μg/ml IVIG were performed in duplicates for each antigen and an average was calculated. Line and error bars represent mean ± SEM for each group. Endpoint tires are expressed as 1/endpoint titre concentration. There were no significant differences in the pre- and post-2009 HA-specific IgG levels.

### Proteins and biotinylation

Influenza HAs and HIV-1 gp140 proteins were sourced from Sinobiological, Shanghai, China. 50μg of protein was biotinylated using EZ-Link® Sulfo-NHS-LC-Biotin kit (Thermo Fisher Scientific, Waltham, MA) following manufacturer’s recommendations. 50-fold biotin excess was used to label each of the HA proteins and a 20-fold excess for the HIV-1 gp140 control protein. ELISA experiments using streptavidin-HRP to assess biotinylation indicated the above levels of biotin showed generally more efficient biotinylation compared to lower levels of biotin (20- and 10-fold excess respectively) and higher amounts of biotin (100- and 50-fold respectively) did not result in improved biotinylation (data not shown). Excess biotin was removed by washing four times with PBS using the 30k Amicon® Ultra-0.5ml Centrifugal Filter Units (Millipore, Billerica, MA).

### Hemagglutination Inhibition (HI) assay

HI titres were measured in either plasma or serum samples as previously described [22]. Briefly, samples were pre-treated with receptor destroying enzymes (Denka Seiken C. Ltd). Plasma or sera were serially diluted 2-fold from 1:10 up to 1:640 in PBS. The capacity of the plasma or sera to inhibit agglutination of 1% turkey red blood cells by specific strains of IAV was measured. Titres are stated as the reciprocal of the highest dilution of plasma or serum where hemagglutination is inhibited.

### IgG ELISA

HA-specific IgG ELISA assay was performed to calculate IgG endpoint titres. 5 HA proteins (H1N1 A/Solomon Islands/03/2006, A/California/4/2009, H3N2 A/Wyoming/03/2003, H2N2 A/Canada/720/2005 and H5N1 A/Vietnam/1194/2004) and SIV gp120 (Sinobiological) were diluted individually at 500ng/ml in PBS. 96-well flat bottom MaxiSorp^TM^ plates (Nalgene Nunc, Rochester NY) were coated with 50ng/well of each relevant protein at 4°C overnight. Plates were washed with 0.05% Tween-20 in PBS, and a final wash of PBS using Wellwash^TM^ Versa microplate washer (Thermo Fisher Scientific). Subsequent incubation steps were performed at room temperature with gently agitation.

For plasma samples, non-specific binding was blocked using 1% fetal calf serum (FCS; Thermo Fisher Scientific) in PBS for 1hr. Wells were washed, 2-fold serial dilutions (1:40 to 1:81,920) of plasma samples in 1% FCS were added, incubated 2 hr and washed. IgG in plasma was detected adding 1:4,000 rabbit anti-human IgG/HRP conjugated (Dako, Denmark) and washed after 1 hr. 100 μl 3,3’,5,5’-Tetramethylbenzidine (TMB; Sigma-Aldrich) was added to develop color and stopped with 100 μl 1M HCl. Absorbance was measured at 450 nm. Each serial dilution was done in duplicate for each antigen and gp120 SIV was included to calculate background of each sample. Endpoint was determined as the well with the lowest concentration of IVIG where the absorbance was higher than gp120 SIV + 3SD. On account of higher background using the above method on IVIG samples, a high-protein blocking buffer was used with these samples (containing 10% FCS, 4% whey protein (Professional Whey, Australia) and 0.5% Tween-20). Half-log serial dilutions of IVIG samples starting at 100 μg/ml were added in duplicates for each antigen. IgG detection and endpoint titre calculations were performed as above. Endpoint titres of IVIG are expressed as 1/endpoint dilution titre μg/ml.

### Functional ADP assay

To evaluate the functional significance of ADP antibodies, we developed an assay for antibody-mediated uptake of live IAVs by THP-1 monocytic cells through intracellular staining for influenza NP. IAV was first incubated with human or macaque IgG for 1 hr at 37°C. In order to measure Fc-mediated phagocytosis rather than natural binding of IAVs to sialic acid, cells were pre-treated with 50ug/ml sialidase (Sigma-Aldrich, St Louis, USA) for 1 hr at 37°C then washed away with cold serum free RPMI. 5x10^5^ THP-1 cells in serum free RPM1 1640 (Gibco, Thermo Fisher Scientific) were seeded in a 96-well u-bottom plate. Cells were then incubated with IAV strains, previously opsonised, or not, with IgG, at a multiplicity of infection of 10 for 1 hr then washed three times with RF10 (RPM1 supplemented with 10% FCS) to remove free virus. Following 6 hr incubation at 37°C, THP-1 cells were washed with FACS wash buffer (2mM EDTA, 1% BSA in PBS), fixed with 4% paraformaldehyde and permeabilized using 0.1% triton-X. Finally, cells were stained with 1:100 anti-NP-FITC antibody (431, Abcam, Cambridge, UK) for 45 min and washed with FACS wash buffer. All cells were acquired immediately on the LSR Fortessa (BD, Franklin Lakes, NJ) and analyzed using FlowJo analysis software version 10.0.7.

### Infected THP-1 supernatant transfer to MDCK cells

**Fig 4:**
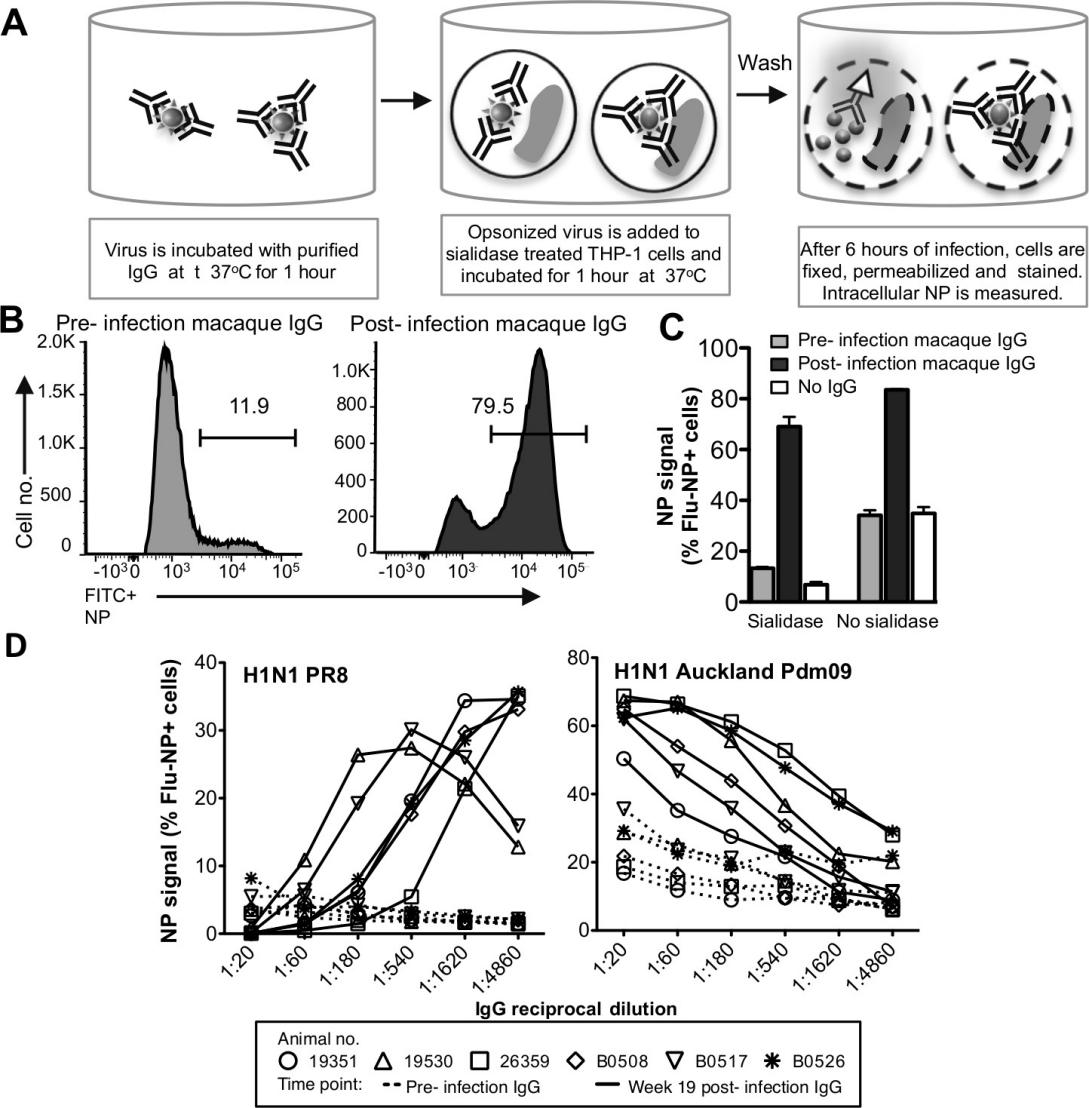
Antibody-mediated uptake of live IAVs. **(A)** Functional ADP assay. Sialidase treated THP-1 cells were infected for 1hr at a M.O.I of 10 with IgG-opsonized IAV, washed and cultured 5–6 hr. Cells were fixed, permeabilized and intracellular NP measured by flow cytometry. **(B)** Gating strategy of NP+ monocytes in the presence of purified macaque (#26359) IgG pre influenza infection (left panel) and at week 19 post influenza infection (right panel). Data is representative of n = 3 independent experiments. **(C)** Uptake rate was compared between sialidase treated or untreated cells in the absence or presence of macaque (#26359) IgG pre and post influenza infection diluted 1:100 using H1N1 Auckland Pdm09 (A/Auckland/1/2009) virus. Data represents mean + SD of three replicates. **(D)** Half log serial dilutions (1:20 to 1:4860) of naïve and PR8-X31 infected macaque IgG were examined in their ability to mediate uptake of a matched IAV (PR8) and mismatched strain (Auckland Pdm09).

We studied the ability of THP-1 cells infected with influenza in an antibody-dependent manner to either release free virus or contain IAV. Sialidase treated THP-1 cells (5x10^5^ cells) and untreated A549 cells (~5x10^5^ cells, a respiratory cell line supportive of influenza infection) were infected with PR8 virus (M.O.I. of 10, in 200 μl) opsonised or not with IgG from influenza-infected macaques. We studied an antibody dilution of 1:1000 since we showed that this dilution retains ADP activity in the absence of neutralization (illustrated in Fig 4D). A 1:1000 IgG dilution results in a comparable concentration of IgG to that used above in the ADP-SHIP assay (purified macaque IgG was 15.9 mg/ml on average). Cells were washed twice with RF10 to eliminate excess virus and antibodies from supernatant. After 20 hr of culture at 37°C, 150 μl of supernatant was transferred to ~5x10^5^ MDCK cells in 150 μl. To assess NP levels of the donor cells, THP-1 and A549 cell were resuspended, fixed, permeabilized, labelled with anti-NP antibody and analysed by flow cytometry as above. NP signal in the recipient MDCK cells was measured 20 hrs after the supernatant transfer by intracellular NP staining as above.

### Patients, IVIG, human plasma and sera samples

ADP in healthy adults was studied in plasma from Australian subjects (*n =* 14) aged between 22–60 years old. To assess ADP in the general population, IVIG preparations manufactured from 2004 to 2010 (*n =* 17) by bioCSL (Parkville, Australia) were tested. To investigate the presence of influenza-specific ADP after influenza infection, we evaluated paired sera samples from three subjects who naturally contracted IVI. IgG from normal human serum (Sigma-Aldrich) was used as positive control in all ADP assays. We purchased influenza seronegative human IgG (MBL Bion, Des Plaines, IL) or used IgG purified from influenza-naïve macaque plasma (pre-infection samples from below) as negative controls.

Total IgG was purified from 50–200μl plasma or serum using Protein G HP Multitrap and Antibody Buffer Kit (both GE Healthcare, UK) according to manufacturer’s instructions. Eluted IgG was washed twice with PBS using 30k Amicon® Ultra-4 Centrifugal Filter Units (Millipore, Billerica, MA). Total IgG concentration was measured by NanoDrop 2000 (Thermo Fisher Scientific, Waltham, MA). IgG from human serum (Sigma-Aldrich, St. Louis, MO) was used as positive control in all ADP assays.

### Macaque plasma samples

We selected plasma from 6 pigtail macaques (*Macaca nemestrina)* infected with IAV in a previous study described by Reece et al [23]. Briefly, animals were inoculated intranasally with 10^8^ PFU twice with both PR8 A(H1N1) A/Puerto Rico/8/1934 and X31 A(H3N2) A/HKx31 viruses that expressed a SIV CTL epitope as part of previous SIV vaccine study [23]. Animals #19530, #19351 and #B0508 were infected at week 0 and 9 with X31, and at week 4 and 17 with PR8. Animals #26359, #B0517 and #B0526 were inoculated with PR8 at week 0 and 17; and with X31 at week 4 and 9. We previously showed that all animals seroconverted to the IAV infections and shed virus was recovered early after infection [23]. Longitudinal plasma samples were assessed to measure influenza-specific ADP pre and post influenza infection.

### Ethics statements

All human subjects provided written informed consent and subjects had the opportunity to withdraw from the study at any time. The studies were specifically approved by the University of Melbourne and Alfred Hospital research and human ethics committees.

Experiments on the macaques were approved by the University of Melbourne and CSIRO Animal Health Animal Ethics Committees, approval number 1315. Samples were from the same ethics committee-approved study previously described by our group [23]. All animals were cared for by dedicated trained animal technicians and humanely euthanized at the end of the experiments with a lethal dose of pentobarbitone IV. No disease or clinical symptoms occurred during the study. Animals were housed at CSIRO animal health and held according to Australian NHMRC animal welfare guidelines in group housing with cages 2.0 m high, 3.0 m long and 1.2 m wide. An environmental enrichment strategy for animals provided toys, treats, fresh fruits and vegetables. Specifically, there were regular changes of equipment and toys, facilities to allow swinging and jumping and engaging in activities from floor to ceiling, wood chip substrates on the floor to provide opportunity for foraging, the use of puzzle feeders, placement of food in different locations in the enclosures and access to partially partitioned areas within the enclosures for rest and privacy. There was also access to mirrors and water pools. They were socially housed in groups. Animals were monitored daily for health and welfare. All procedures were carried out with sedated animals. Animals were anesthetized intramuscularly with ketamine (10 mg/kg) prior to any procedures.

### Data analysis

Statistical analysis was performed using Prism GraphPad v5 (GraphPad Software, San Diego, CA). Data were analyzed by one-way ANOVA followed by a Bonferroni’s comparison (Fig 2B), two-way ANOVA together with a Bonferroni comparison (Fig 3A–3C) and Pearson’s product-moment correlation coefficient (Fig 2C and 2D). ADP values were considered positive when above mean + twice SD for HIV-1 gp140.

## Results

### Influenza-specific ADP assay

We modified an ADP assay previously developed to study HIV-specific immunity [19] to measure the ability of antibodies to mediate internalization of fluorescent particles coated with influenza HA protein. The assay is illustrated in Fig 1A, where influenza seronegative human serum IgG, seronegative macaque plasma IgG, plasma IgG from influenza seropositive healthy adults, and plasma IgG from macaques experimentally infected with influenza are studied (representative plots in Fig 1B and 1C). When the phagocytic THP-1 monocytic cells were incubated with influenza seronegative samples, 10–11% of cells internalized at least one HA-coated bead (right column, Fig 1C). In contrast, 42–49% of cells internalized one or more beads when incubated with the influenza seropositive human or macaque samples. Thus, anti-HA antibodies from influenza-immune sera/plasma mediate uptake of HA-beads.

### Influenza-specific ADP in healthy human donors

To probe the ability of adult sera to mediate influenza-specific ADP we studied serum IgG from 14 healthy, HIV-negative Australians during 2013–2014. As expected, the majority of donors had HI-inducing antibodies to a variety of H1N1 strains, as well as a H3N2 strain but not to H5N1 or H7N9 strains (Fig 2A).

Serum IgG from all but one of the 14 subjects had appreciable ADP activity to recently circulating H1 proteins and H3 protein (Fig 2B, left panel). We then evaluated ADP against HA proteins from non-circulating H2, H4, H5 and H7 IAV subtypes (Fig 2B, right panel). All subjects had detectable ADP against both H5 and H2 HA proteins, but at generally lower levels than that observed to H1 and H3 proteins. A subset of subjects also had weak ADP to a H4 protein (6 out of 14) and H7 protein (9 out of 14). Across the group, ADP was significantly higher to the H2 and H5 HA tested proteins than to the negative control HIV gp140 (both P < 0.0001).

The detection of ADP activity to non-circulating strains in the absence of HI activity to the same strain, suggests that the antibodies are cross-reacting with shared epitopes induced by circulating strains. To evaluate this further, we correlated the HI titres with ADP activity to the HA protein from the same virus and found significant correlations (Fig 2C). To study the relationship between total HA-specific antibodies (rather than the subset of Nabs) with ADP activity we also performed IgG ELISA assays across 5 of the HA proteins studied. We found positive correlations between HA-specific IgG levels and ADP activity for all 5 HA proteins studied (Fig 2D). Overall this suggests that exposure to seasonal influenza strains (via infection or vaccination) is generating a subset of HI-inducing and total HA-specific antibodies that can cross-react with non-circulating IAVs and mediate ADP.

### ADP to influenza in IVIG preparations

To study sera across a large population base, we analyzed IVIG for ADP activity. Studying IVIG before and after the spread of the H1N1pdm during 2009 allowed us to evaluate whether cross-reactive ADP activity to H1N1 Pdm09 was prior to 2009. Indeed, ADP activity to the HA protein of H1N1 Pdm09 was observed in all 8 IVIGs prepared prior to 2009 (Fig 3A). There was a substantial rise in ADP to H1 Pdm09 in IVIGs prepared post 2009, consistent with a subset of the donors boosting their ADP activity to this protein following H1N1 Pdm09 infection. The rise of H1 Pdm09-specific ADP was consistent across multiple experiments (illustrated in Fig 3A and 3B), as was background ADP levels to HIV-1 gp140. Interestingly, levels of ADP to seasonal H1N1 and H3N2 HAs were modestly but significantly higher in IVIG preparations made post 2009 (Fig 3B), suggesting exposure to H1N1 Pdm09 may have broadened ADP activity.

We next evaluated whether ADP to HA proteins from rarely circulating IAV subtypes was present in IVIG samples (Fig 3C). We detected substantial levels of ADP to H2 and H5 HA proteins, low level ADP to a H4 HA and negligible ADP to a H7 HA protein. IVIG preparations made post 2009 had modestly but significant higher levels of ADP to H2, H4 and the H7 HA proteins tested (Fig 3C).

To evaluate whether the small increase in ADP activity observed in post-2009 IVIG samples was also reflected in levels of total HA-specific IgG levels, we performed IgG ELISA assays on the 17 IVIG samples to a range of HA proteins (Fig 3D). Although there was a slight increase in H1pdm-specific IgG levels in post-2009 samples, we did not observe an increase in HA-specific IgG levels to other HA proteins and none of the differences were statistically significant. This suggests that H1N1pdm infections amongst the IVIG donors led to an overall enhanced functional HA-specific ADP response without a significant increase in total HA-specific IgG levels.

### Influenza-specific ADP after experimental influenza infection of macaques

**Fig 5:**
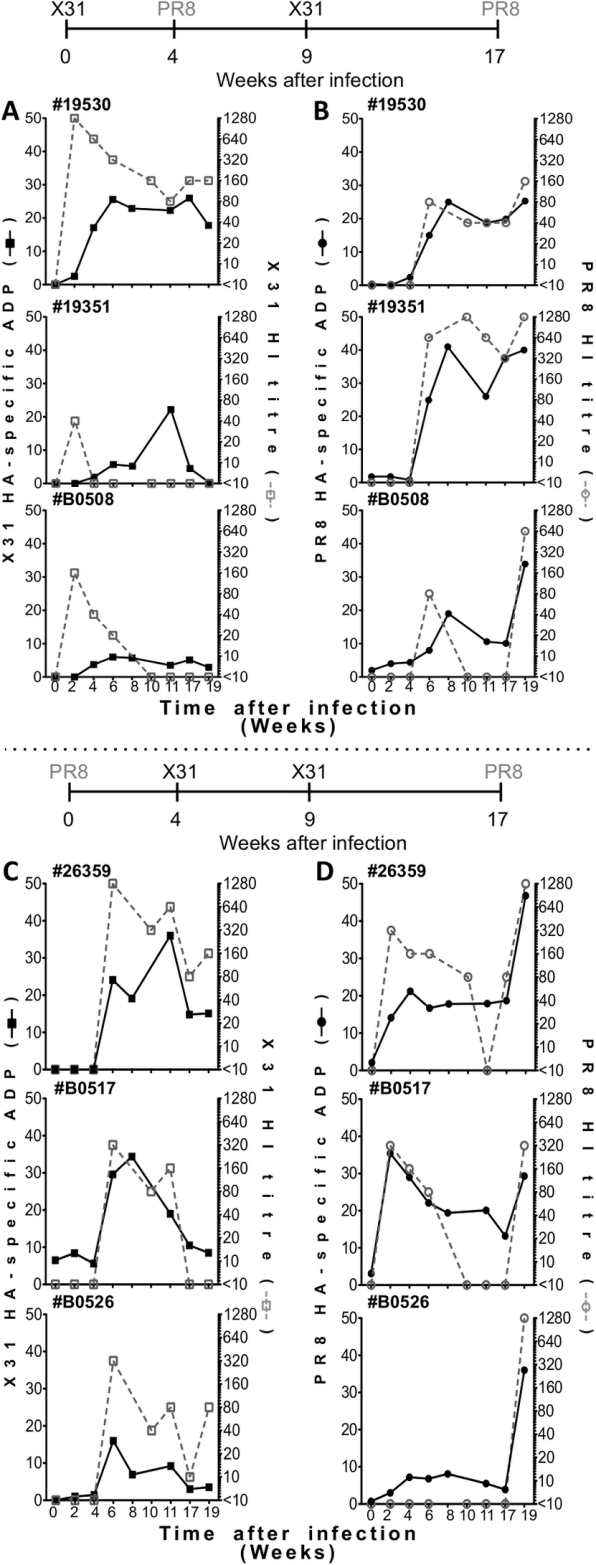
ADP in pigtail macaques infected with IAVs. HI titres (grey open symbol; dotted line) and ADP activity (black filled symbol; solid line) of 6 pigtail macaques infected twice with X31 (H3N2 A/HKx31) and PR8 (H1N1 A/Puerto Rico/08/1939). Grey arrows indicate influenza strain and time of infection which is summarized above the panels–the macaques in the top panels (A, B) were first infected with X31 and macaques in the bottom panels were first infected with PR8 (C, D). X31 and PR8 immune responses for each macaque are illustrated on A, C and B, D respectively. All ADP responses were corrected subtracting influenza-naïve macaque (mean of 3 animals) background.

Analysis of influenza-specific antibodies in adult humans is complicated by the unknown number of previous exposures to influenza. We therefore studied sera samples from 6 macaques experimentally infected serially with H1N1 (PR8) and H3N2 (X31) strains from a previously trial [23]. Infections with PR8 or X31 are indicated with arrows in Fig 5. We previously reported the animals seroconverted by HI and shed IAV in their respiratory tracts [23]. For each macaque we studied ADP levels and HI titres to both X31 (Fig 5A and 5C) and PR8 (Fig 5B and 5D).

Overall, ADP activity tended to mirror HI activity. The onset of ADP activity generally occurred simultaneous to the HI antibodies and was to the homologous infecting influenza subtype. That is, when X31 was the initial infection, X31 ADP and HI occurred earlier than PR8 ADP and HI (Fig 5A and 5B) and vice versa when PR8 was the initially infecting virus, PR8 ADP and HI occurred first (Fig 5C and 5D). However, there were instances when HI activity was detectable earlier after infection than ADP activity, particularly to X31 after an initial infection with X31 (Fig 5A). In several animals, weak but detectable ADP activity persisted when HI antibodies were no longer detected (e.g. Fig 5A and 5C middle panels to X31 and Fig 5B lower, D middle and lower panels to PR8). This may reflect higher levels and/or lower decay rates of ADP-antibodies compared to HI antibodies.

### Antibody-mediated uptake and clearance of live IAVs

The above studies analyzed the phagocytosis of HA-coated fluorescent beads which may not perfectly represent the phagocytosis of whole live IAVs. We studied sialidase-treated THP-1 cells for uptake of opsonized IAV. We measured intracellular NP levels after 6 hours by flow cytometry as a read out of virus uptake (illustrated in Fig 4A). An example of Auckland Pdm09 virus (H1N1 A/Auckland/1/2009) opsonized with pre and post infection macaque sera IgG shown in Fig 4B, which shows high levels of intracellular NP only within THP-1 cells exposed to post-infection sera IgG. The rise in uptake of opsonized live IAV (1:100 serum IgG dilution) by THP-1 cells was consistent across all 6 macaque IgG samples studied, with a mean fold increase in viral NP detection of 3.1 (range 1.8–5.1 fold increase) when post infection IgG is used. As expected, sialidase treatment reduced the background uptake of IAVs through sialic acid either in the absence of IgG or in the presence of pre-infection macaque IgG (white and gray bars in Fig 4C). This provides evidence that this functional ADP assay primarily measures phagocytic uptake of IAV mediated by antibodies rather than other receptor mediated entry. The effect of sialidase on influenza-uptake of THP-1 cells in the presence of pre-infection macaque IgG was consistent across the 6 macaques studied, with a mean fold reducing in NP signal of 2.1 (range 1.7–2.7).

We utilized this functional live IAV phagocytosis assay to study serum IgG samples from all 6 macaques before and after IVI. We studied phagocytosis to both one of the homologous infecting viruses (PR8) and a heterologous H1 virus (H1N1 Pdm09; A/California/04/2009; Fig 4D). Prior to infection, serum IgG mediated minimal uptake of the homologous PR8 virus in all 6 macaques. Post-infection IgG showed low levels of virus uptake at high levels of IgG but as the IgG was titrated out to low levels (>1:540 dilution), there was substantial virus uptake and NP^+^ THP-1 cells in all animals. This is consistent with homologous PR8-specific NAbs blocking virus uptake at low IgG dilutions. The phagocytosis-mediating antibodies are still present at higher dilutions and mediate uptake of the homologous virus as the NAbs are diluted out.

Such an effect should not of course be seen in the absence of NAbs so we also analyzed NP signal of sialidase treated THP-1 cells with opsonized H1N1 Auckland Pdm09 (Fig 4D right panel). Compared to PR8 infection, background NP levels in THP-1 cells were higher with Auckland Pdm09 infection when IgG from influenza naïve macaques was studied, presumably reflecting the greater sensitivity of THP-1 infection to this H1N1 Pdm09 strain. However, there was a substantial increase in antibody-mediated virus uptake in all 6 post-infection IgG samples. There was no inhibition of uptake at high IgG concentrations, as was seen with PR8, consistent with the lack of heterologous NAbs. Thus, this functional ADP analysis showed substantial cross-reactive ADP antibodies to H1N1 Pdm09 capable of mediating virus uptake into the monocytic cell line in sera IgG from macaques only infected with PR8 and X31 strains. It is notable that, although present, these H1N1 Pdm09-specific ADP antibodies titrated out more rapidly than the PR8-specific ADP antibodies.

### Clearance of opsonized IAV by THP-1 cells

**Fig 6:**
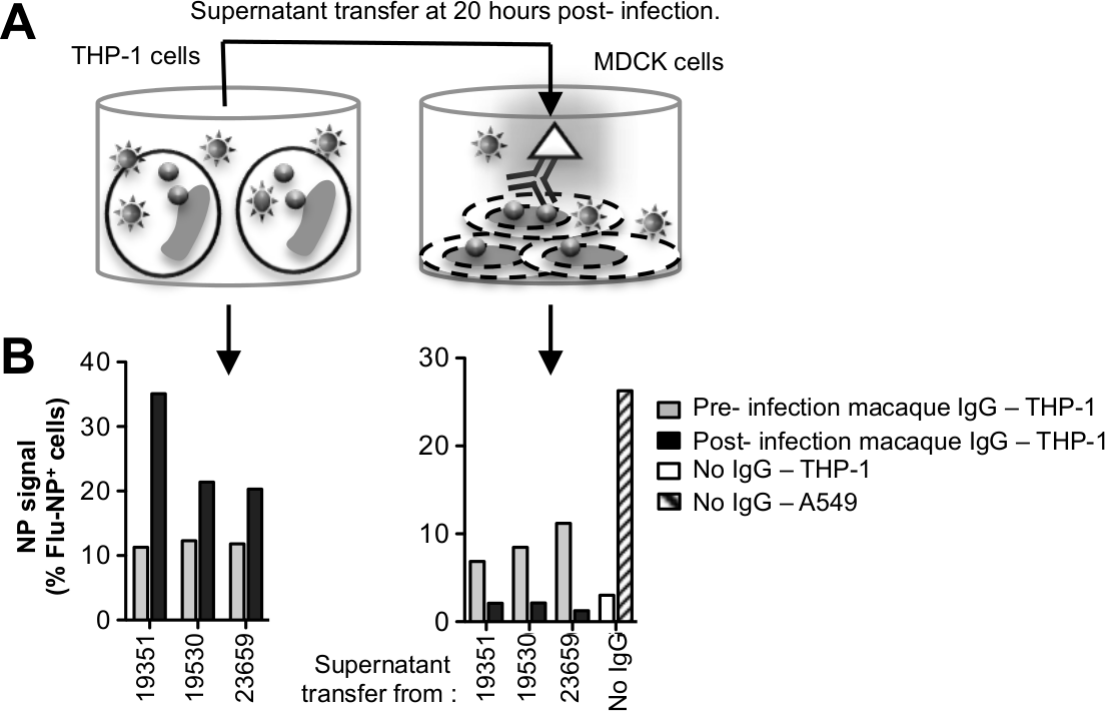
Influenza virus is not released from THP-1 cells after antibody-mediated virus uptake. **(A)** The assay setup. Sialidase treated THP-1 cells and untreated A549 cells were infected with PR8 virus (M.O.I. of 10). Virus was previously opsonised or not with IgG from either naïve or influenza infected macaques. IgG was diluted 1:1000 to avoid neutralization of virus. Cells were washed twice to eliminate excess virus and antibodies from supernatant. After 20 hr of culture at 37°C, supernatant was transferred to MDCK cells. THP-1 pellets and resuspended A549 cells were fixed, permeabilized, labelled with anti-NP antibody and analysed by flow cytometry. 20 hr later, infection rate in MDCK was measured with intracellular NP staining. **(B)** Confirms antibody-mediated uptake of the PR8 virus by the THP-1 cells using sera post PR8 infection. **(C)** Shows the ability of supernatant from the antibody-mediated infection of THP-1 cells, or control THP-1 or A549 cells in the absence of antibody, to infect MDCK cells. Data are representative of n = 3 independent experiments.

The above studies showed substantial Ab-mediated virus uptake into THP-1 cells. However Fc-receptor bearing cells such as monocyte/macrophages are not generally supportive of high levels of IAV release [24–26]. To formally assess whether the THP-1 cells can take up IAV in an Ab-dependent manner but not release progeny virus to infect other cells, we transferred supernatants from THP-1 cells infected with the heterologous H1N1 Auckland Pdm09 virus for 20 hours onto susceptible MDCK cells (illustrated in Fig 6A). This 20 hr infection assay (as compared to the 6 hr assay described above) also showed higher levels of NP detection in THP-1 cells when IgG from macaques after influenza infection was studied (Fig 6B left panel). However, almost no viable virus was released into the supernatants over this time frame, as shown by the low NP levels of MDCK cells using the supernatants from the infected THP-1 cells (Fig 6B, right panel). As a positive control, we showed that supernatants from infected A549 cells resulted in a robust infection of MDCK cells as expected.

### ADP in serum IgG from humans recently infected with H1N1 Pdm09

**Fig 7:**
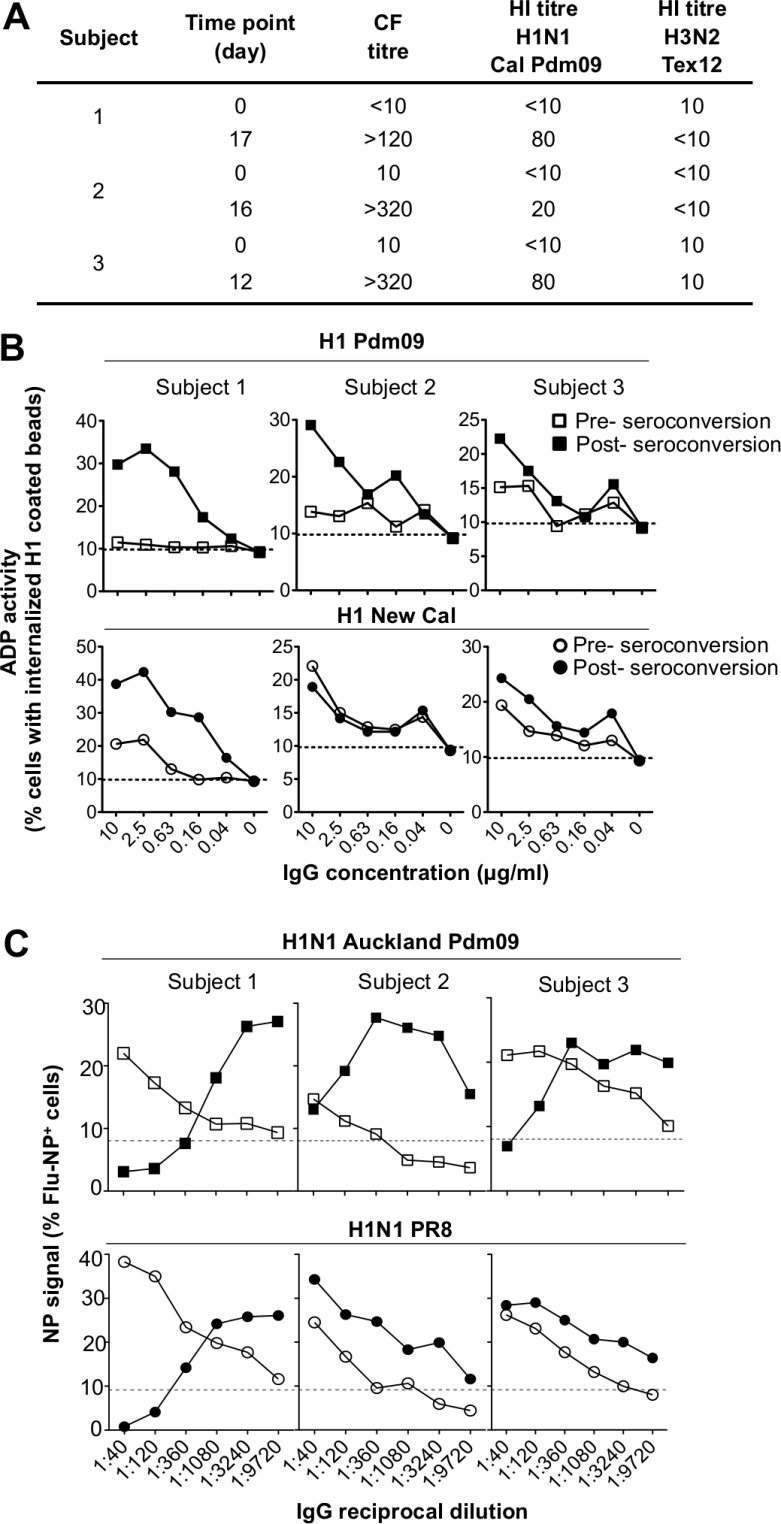
ADP in three humans with symptomatic H1N1 Pdm09 infection. **(A)** Serologically characteristics for early and late time points after an influenza-like illness. **(B)** Comparison of ADP titres for the 3 subjects pre (filled symbol) and post (open symbol) influenza seroconversion of HA-coated beads from A/California/04/2009 (H1 Pdm09, squares) and A/New Caledonia/20/1999 (H1 New Cal, circles). Dotted line represents negative control ADP for an influenza naïve macaque. **(C)** Ability of antibodies to mediate uptake of live IAV was assessed using half log serial dilutions of IgG pre and post seroconversion (1:40 to 1:9710) as for Fig 4. Infection was compared using a matched virus strain to the infection (H1N1 Auckland Pdm09) and a mismatched strain (H1N1 PR8). Dotted line represents uptake of live virus in the absence of IgG.

To study humans naturally infected with influenza, we obtained paired sera samples from 3 adults with probable recent H1N1 Pdm09 infection. All subjects had an influenza-like illness, a rise in complement-fixing (CF) antibodies to IAV, and a rise in HI titre to H1N1 Pdm09 in the absence of a rise of HI titre to other circulating influenza strain (Fig 7A).

We performed ADP assays on the paired serum IgG samples to H1 Pdm09 and a seasonal H1N1 New Cal HA protein first using the bead-based assay (Fig 7B). In all 3 subjects ADP to H1 Pdm09 was low in the pre-seroconversion sample but rose substantially in the sample post infection (Fig 7B, upper panel). ADP to H1N1 New Cal HA protein was higher at baseline in all 3 subjects, consistent with the circulating nature of this virus prior to the circulation of the H1N1 Pdm09 virus (Fig 7B). Interestingly, in Subject 1 and to a lesser extent in Subject 3, there was also a boost in ADP to this seasonal HA tested (H1 New Cal) after the H1N1 Pdm09 infection, consistent with the stimulation of cross-reactive ADP antibodies by the H1N1 Pdm09 infection.

The above work studied the uptake of HA-coated beads in humans recently infected with H1N1 Pdm09. We then studied the ability of these paired sera IgG to mediate ADP of homologous (H1N1 Pdm09) and heterologous (PR8) live IAVs in the functional assay developed for the macaque studies in Fig 4. For the H1N1 Pdm09 (Auckland Pdm09) virus, we saw a low level of functional ADP prior to seroconversion that was substantially boosted after seroconversion (Fig 7C top panels). As for the homologous virus studies in the macaque sera experiments in Fig 4, we saw inhibition of infection in the presence of homologous NAbs (out to dilutions of 1:360–1080) but high levels of ADP out to dilutions of 1:9720. For the heterologous H1N1 virus PR8, all 3 subjects had levels of functional ADP that were boosted by the H1N1 Pdm09 infection (Fig 7C, lower panels). This illustrates that cross-reactive functional ADP are generated in humans following influenza infection. In subject 1, the subject with the highest levels of baseline ADP activity to both H1N1 Pdm09 and PR8, we also observed that PR8-specific ADP was inhibited at high sera concentrations. This suggests that cross reactive PR8-specific NAbs also developed following the H1N1 Pdm09 infection, as has been previously observed in mice [27, 28].

## Discussion

Influenza infection remains a significant disease affecting worldwide health. The presence of specific influenza NAbs consistently correlates with efficacy of viral clearance [29–34]. The role of non-NAbs to IAVs that mediate antiviral functions through Fc receptors on innate immune cells is not yet fully understood. Increasing evidence points to a role for ADCC antibodies, acting via CD16 Fc receptors, in assisting in the control of influenza infection [10, 13, 14, 16, 35, 36]. However, to our knowledge, there has only been one brief report investigating ADP antibodies acting via CD32 receptors in mice [18] and no previous reports in humans or non-human primates. We modified a HIV-specific ADP assay to measure influenza HA-ADP and found that influenza HA-specific ADP was readily detected to multiple HA subtypes in both sera IgG from healthy adults and in IVIG preparations. Of particular interest is the ADP to the avian HA protein H5 and H7 since these viruses are considered to have a high pandemic potential. We speculate that pre-existing ADP (as well as ADCC [10, 13]) to H5 could assist in partial control of infection with this virus. The presence of ADP in IVIG towards H5 and other non-circulating strains such as H2 and H4 suggests that studies are warranted to assess a potential role for existing IVIG preparations in assisting in the control of future pandemics.

Our observations raise the question as to how cross-reactive ADP antibodies develop? Our studies of humans and macaques infected or vaccinated with IAVs offers some insights. In IVIG preparations prior to, and after, the 2009 H1N1 Pdm09 pandemic there was an expected rise in H1 Pdm09-specific ADP. However, there was also a small but significant rise in ADP to many of the heterosubtypic HA proteins tested. Natural infections of humans with H1N1 Pdm09 resulted in a rise in functional ADP at 2–3 weeks to not only H1 Pdm09 but also to other H1N1 viruses. If should be cautioned however that we have studied only a limited number of subjects with H1N1pdm acute influenza infection to date and the initial sera IgG sample was during the early infection phase. In sera IgG from both experimentally infected macaques and naturally infected humans we observed that uptake of live heterologous IAVs into the monocytic cell line was increased. We speculate that the multiple prior lifetime exposures of human adults to IAVs permits a scenario where low-level cross-reactive ADP responses can be boosted by subsequent heterologous infections.

Our studies do not imply that ADP-inducing antibodies are protective against influenza infection and future *in vivo* studies are needed to assess this. Definitive studies are difficult at present in the absence of influenza-specific monoclonal antibodies with only ADP function. Our studies on antibody-mediated live IAV uptake into a monocytic cell line suggests that these cells are capable of clearing influenza *in vitro*. Further development of ADP assay using primary monocyte/macrophages will be of interest. We acknowledge, however, that by mediating enhanced influenza infection, ADP could potentially be harmful in some influenza infection settings, particularly if the strains can replicate efficiently in CD32+ cells or if there is high level CD32 expression on non-immune cells in the respiratory tract. The isolation or engineering of human monoclonal antibodies that mediate only particular functions (neutralization and/or ADCC and/or ADP) and their study in Fc-receptor engineered mice or in macaques should assist further in elucidating a role for functional antibody responses in controlling influenza infection. We also note that complement fixation functions and other antibody functions could also contribute to the control of influenza infection.

It is not known if available or investigational influenza vaccines induce specific ADP activity. Humans with multiple exposures to IAVs may have low-level ADP responses that can be boosted by even suboptimal vaccines. Recent evidence suggests that influenza vaccination tends to primarily boost pre-existing B cell responses [37]. We are currently studying whether standard vaccines can boost or broaden influenza-specific ADP responses. Our data in the report highlight a difficulty in assessing functional ADP assays, since homologous neutralizing antibodies compete to prevent influenza virus phagocytosis when high antibodies are studied.

In conclusion, we characterized influenza-specific ADP activity in the serum IgG from humans and macaques exposed to IAV. We detected broadly cross-reactive ADP responses in healthy humans and in pooled human sera IVIG preparations. Further analysis of the protective capacity of ADP responses against IAV is warranted.
